# Emergency Physicians’ Experience-Remuneration (E-R) Mismatch: A Canadian Healthcare Irony

**DOI:** 10.7759/cureus.41891

**Published:** 2023-07-14

**Authors:** Mohammed Abrahim

**Affiliations:** 1 Division of Emergency Medicine, Department of Family Medicine, McMaster University, Hamilton, CAN

**Keywords:** e-r mismatch, global healthcare systems, canadian health care, physician remuneration, physician training, emergency medicine physician

## Abstract

Conventional wisdom suggests that in almost every profession, the most experienced and educated employees are remunerated at a higher rate than the less experienced ones. For example, new-graduate hires most commonly start at the bottom of the pay scale. No profession could reflect the importance of experience and the need for mastery of skills more than emergency medicine (EM), where a split-second decision could mean the difference between life and death. In Canada, however, EM physicians are remunerated as per a common pay scale that does not consider the length of their education, training, or years of practice. Such an unfair experience-remuneration mismatch (E-R mismatch) could lead to job dissatisfaction, burnout, and switching to other specialties. Given the current EM physician shortage in Canada, the E-R mismatch among such physicians could negatively impact patient care and the health system as a whole and prolong the already long wait times. The aim of this editorial is to shed light on this flaw in the Canadian healthcare system and lead to change toward a fair pay system. The creation of a professional and experience-based hierarchy among Canadian EM physicians should be considered a matter of urgency for those developing health-related legislation.

## Editorial

Emergency medicine certification in Canada

Emergency medicine (EM) is a frontline, high-pressure medical specialty that manages various life-threatening emergencies in the Canadian population. In Canada, EM is a relatively new specialty; it was recognized as a specialty by the Royal College of Physicians and Surgeons of Canada (RCPSC) in 1980 [1]. The first Royal College certifications were first granted in 1983 [1]. To practice EM in Canada, there are two main pathways. The first and most common pathway is for family physicians and is offered by the College of Family Physicians of Canada (CFPC). Prior to 1990, medical students who graduated and completed general medicine internship rotations practiced EM without the requirement for dedicated EM training [2]. In 1966, the Certification in the College of Family Physicians (CCFP) was created, but it was not until the early 1990s that it became a mandatory requirement for family physicians. A two-year residency training program in family medicine is considered a sufficient qualification to practice EM in Canada [1]. The second certification is also provided by the CFPC as an added competence in EM, which could be obtained by two options: either by challenging the certification exam after practicing EM as a family physician at a rate of at least 400 hours per year for the four-year period immediately prior to the application (practice-eligible) or through an additional year of training in EM, as well as successfully passing the EM exam to obtain the Canadian College of Family Physicians Emergency Medicine (CCFP-EM) certification [2]. The second pathway, provided by the RCPSC, is a five-year EM residency training program with certification as a Fellow of the Royal College of Physicians of Canada (FRCPC) [2].

Remuneration

The Canadian healthcare system is a one-tier, universal, public system funded by tax revenue [3]. Approximately 70% of the money spent on healthcare comes from general taxation. The 10 provinces and three territories receive healthcare funding from the federal government [3]. The majority of EM physicians are paid as “independent contractors” and get paid primarily either by a fee-for-service (FFS) payment model, where EM physicians submit claims to their local Ministry of Health using billing codes that take into account the acuity, duration, and timing of the encounter, in addition to any billable procedures. The alternative funding plan is another payment model where physicians are often paid an hourly rate and a certain percentage of FFS billings [4]. Only in the province of Quebec are FRCPCs paid slightly more than CCFP-EMs. However, the number of specialist (FRCPC) positions in each region is limited by work permits [1]. While CCFPs need only two years of residency and CCFP(EM) could be obtained with and without a third year of residency training, FRCPCs require five years of EM residency [2]. Therefore, in all provinces, CCFPs could begin earning full EM physician wages three years and one year prior to their FRCPC and CCFP(EM), respectively [2]. Ironically, and unique to Canada, the EM physicians with fewer years of training end up being paid more.

Although there is no lack of news coverage on physician pay in Canadian media outlets, the discussion on the topic of E-R mismatch is almost non-existent. The public is generally unaware that EM physicians are devoid of retirement payments, extended health benefits, or sick leaves. The public is also unaware that the published income of a physician is the gross income and does not take into account physicians' business expenses, staff salaries, continued medical education including conferences, liability insurance, and several regulatory bodies' dues and memberships, in addition to the cost of the years of pre-medical and medical school student debt.

EM practice in Canada

There has been a chronic and increasing physician shortage in Canada that persists not only among EM physicians but also among family physicians [4]. Currently, EM physicians are at increased risk of experiencing stress and burnout not only compared with other specialties but also to other emergency medical staff [4]. EM job stress appears to be cumulative, leading to higher burnout and stress among more experienced EM physicians, which can lead an individual to leave the profession. EM physicians have a higher probability of leaving their specialties than other specialties and have a significantly decreased survival after 10 years [4]. Circadian rhythm disruption, secondary to shift work, high stress, and burnout, leads to a variety of compensatory unhealthy behaviors, such as junk food consumption and addiction to drugs (including stimulants) or excessive alcohol consumption [4]. Furthermore, EM physicians are more likely to leave their field when they are fully experienced [4]. Because the need for EM physicians exceeds supply, the majority of physicians practicing EM in Canada are not EM physicians but family physicians [1]. However, there is also a severe shortage of family physicians, adding more stress and strain on Canadian emergency departments (EDs).

Moreover, staff shortages lead to longer wait times and subsequently patient frustration and escalating violence against ED staff [5]. Because EM physicians are independent contractors, they are not covered by workplace insurance, despite being the healthcare frontline with the highest risk of exposure to violence, infections, and stress. The aforementioned violence against EM physicians increases the risk of mental health ailments, including stress, depression, anxiety, and suicidal ideation [4]. The absence of a clear plan to prevent or manage violence against EM physicians could lead to the perception of a lack of help when being attacked in the ED. In turn, this may lead to the underreporting of such events when violence against EM physicians is considered a part of the job [4]. Senior EM physicians may seek early retirement and/or change specialty, move to administrative positions, or leave the medical field entirely [5].

Conclusions

It is suggested herein that Canadian policymakers and healthcare managers should establish an equitable experience-based compensation for Canadian EM physicians that takes into account physicians' invaluable experience and creates a professional hierarchy. Fixing the current E-R mismatch could promote job satisfaction, reduce intent to leave the specialty, increase subsequent EM career longevity among experienced EM physicians, and prevent (or at least reduce) the rate of migration to countries that value their expertise. It could also affect patient care by reducing wait times, reducing medical errors, and increasing the quality of healthcare provided. More importantly, with increasing rates of violence against EM physicians, the implementation of Canada-wide workplace insurance coverage for EM physicians akin to other EM providers should be introduced.

Finally, increasing the number of EM residency spots, to address the shortages and increase the number of EM-trained physicians, should be considered. In addition, both the CFPC and RCPSC should adopt uniform EM training programs and certification criteria to standardize the practice of EM in Canada; this appears long overdue and will work to unite the specialty of EM under one discipline. By making these changes, the present culture, where an EM career is not a lifelong career but rather a specialty for the young with limited experience, can be changed. Increased compensation could be financial but also take alternative forms, such as fewer work hours by being remunerated more per hour and working fewer night shifts.
